# Proteomic analysis of protein expression profiles during hyperthermia-induced apoptosis in Tca8113 cells

**DOI:** 10.3892/ol.2013.1354

**Published:** 2013-05-20

**Authors:** WEN JIANG, LI BIAN, NING WANG, YONGWEN HE

**Affiliations:** 1 Department of Dental Research, The Affiliated Stomatological Hospital of Kunming Medical University, Kunming, Yunnan 650031;; 2 The First Affiliated Hospital of Yangtze University, Jingzhou, Hubei 434000;; 3 Department of Pathology, The First Affiliated Hospital of Kunming Medical University, Kunming, Yunnan 650032, P.R. China

**Keywords:** proteomic, hyperthermia, apoptosis, cancer, mass spectrometry analysis

## Abstract

The aim of the present study was to explore protein expression profiles during cancer cell apoptosis induced by hyperthermia. A hyperthermia-induced apoptosis model was established using a Tca8113 cell line derived from a human tongue squamous cell carcinoma, which underwent fluorescent differential display two-dimensional (2D) gel electrophoresis at 2, 6, 8, 12 and 24 h following the induction of hyperthermia. Proteins were identified by mass spectrometry analysis. Expression changes in the proteins were detected by western blot analysis. A total of 107 proteins were detected that exhibited different expression levels in the hyperthermia-treated cells compared with the controls, and 57 of these proteins were identified. Expression changes in the representative proteins were further verified by western blot analysis. These 57 proteins were identified according to the following functional groups: energy metabolism-related enzymes, cytoskeleton-related proteins, chaperones, transcription factors, protein synthesis-related proteins and cell division- and proliferation-related proteins. These groups included 44 upregulated and 13 downregulated proteins. Among the 44 upregulated proteins, 27 were upregulated continuously, eight were upregulated at an early time-point and nine were upregulated at a middle to late time-point. Among the 13 downregulated proteins, five were downregulated continuously, six were downregulated at an early time-point and two were downregulated at a middle to late time-point. These results indicate that hyperthermia-induced Tca8113 cell apoptosis is controlled by multiple factors, which include time and regulatory proteins.

## Introduction

Malignant tumors are treated with a combination of therapeutic modalities, including surgery, radiotherapy, chemotherapy, immunotherapy and hyperthermia therapy. Hyperthermia is effective in killing tumor cells; moreover, it is capable of increasing the sensitivity of tumor cells towards radiotherapy and numerous antitumor drugs (1). Therefore, hyperthermia is considered to be an effective and promising tumor therapy (2). Hyperthermia inhibits tumor cells through the five following mechanisms: i) by inhibiting tumor cell respiration, thereby increasing anaerobic glycolysis and increasing the acidity of the environment, which may result in damage to the tumor cell membranes and the release of lysosomal acid hydrolase; ii) by altering the enzyme structure of the cytoplasm and the nucleus, thereby leading to a metabolic disorder; iii) by inhibiting the synthesis of DNA, RNA and the associated proteins, thereby leading to the aggregation of denatured nuclear matrix proteins; iv) by impairing the cytoskeleton; and v) by inhibiting anti-apoptotic proteases and/or the activity of oncogenes, and enhancing pro-apoptotic proteases and/or tumor suppressor gene activity, thereby leading to tumor cell necrosis and/or the induction of apoptosis (3–6).

Hyperthermic temperatures between 40 and 45°C kill tumor cells, mainly by inducing apoptosis (7,8). The therapeutic temperature for treating an oral tumor is 43°C, which is used to treat malignant oral tumors by inducing tumor cell apoptosis. Hyperthermia-induced tumor cell apoptosis relies on numerous approaches and regulation factors. It has been demonstrated that hyperthermia is able to activate the release of pro-apoptotic factors, such as cytochrome *C*, apoptosis-inducing factor (AIF) and Smac/Diablo, from the mitochondria, and activate downstream effectors to induce apoptosis (9). In addition, hyperthermia has been demonstrated to enhance Fas-L, tumor necrosis factor-α (TNF-α) and TNF-related apoptosis-inducing ligand (TRAIL) expression, and to increase tumor cell sensitivity towards their receptors, which ultimately induces apoptosis through the death receptor pathway (10–12). Furthermore, it has been indicated that hyperthermia induces intracellular reactive oxygen species (ROS) production and the activation of downstream effectors, which leads to apoptosis (13,14). The treatment also promotes the influx of extracellular Ca^2+^ and/or the damage of the cytoplasmic calcium pool, thereby increasing intracellular Ca^2+^ concentrations and activating Ca^2+^-related enzymes, thus resulting in apoptosis (15,16). Moreover, it has been demonstrated that hyperthermia is able to activate or upregulate the gene or protein expression of p53, Bax, Bak and caspase family members, again leading to apoptosis (9,17–20). During hyperthermia-induced tumor cell apoptosis, anti-apoptotic protective factors are produced by the tumor cells for the maintenance of self-survival and for protecting the cell from damage. For example, heat shock proteins (HSPs) are produced under stress to maintain cell survival (21). It has been demonstrated that the expression of HSPs is increased significantly following hyperthermia (22–25), which may inhibit apoptosis. Additionally, it has been demonstrated that Bcl-2 and Mcl-1 are also able to protect tumor cells from hyperthermic damage (26,27). However, the mechanism of hyperthermia-induced tumor cell apoptosis has not been fully elucidated.

In our previous study, we demonstrated that hyperthermia-induced Tca8113 cell apoptosis involved changes in the expression and function of multiple proteins (28). In the present study, a hyperthermia-induced apoptosis model was established using the Tca8113 cell line derived from a human tongue squamous cell carcinoma. Cell lysates were subjected to fluorescent differential display two-dimensional (2D) gel electrophoresis 2, 6, 8, 12 and 24 h after hyperthermia treatment. A total of 107 proteins were detected that exhibited different expression levels in the hyperthermia-treated cells compared with in the controls, using matrix-associated laser desorption/ionization (MALDI)-time of flight (TOF) or MALDI-TOF/TOF mass spectrometry analysis. This method allowed us to obtain the peptide mass fingerprint, and 57 proteins were identified by searching the SwissProt and NCBInr databases. Following the analysis of the protein profiles, the results indicated that hyperthermia-induced Tca8113 cell apoptosis is controlled by multiple factors, including time and regulatory proteins.

## Materials and methods

### Materials

The human tongue squamous cell carcinoma cell line, Tca8113, was purchased from Shanghai Cell Bank (Chinese Academy of Sciences, Shanghai, China). The CyDye Difference Gel Electrophoresis (DIGE) fluorescent Cy2, Cy3 and Cy5 were purchased from GE Healthcare (Uppsala, Sweden). The heat shock 70 kDa protein (HSP70; D69), stathmin 1 and Lamin A/C polyclonal antibodies were purchased from Cell Signaling Technology, Inc. (Danvers, MA, USA). Cofilin 1 (5) sc-53934 mouse monoclonal IgG_2b_ was purchased from Santa Cruz Biotechnology, Inc. (Santa Cruz, CA, USA). The anti-phosphoglycerate mutase 1 (PGAM1) antibody (rabbit polyclonal to PGAM1; ab96622) and the anti-Δ(3,5)-Δ(2,4)-dienoyl-CoA isomerase mitochondrial (ECH1p) antibody (rabbit polyclonal to ECH1p; ab90645) were purchased from Abcam (Cambridge, UK).

### Cell culture and hyperthermia treatment

The Tca8113 cells were cultured in RPMI-1640 medium containing 10% fetal bovine serum (with 1×10^5^ U/l penicillin and 1×10^2^ mg/l streptomycin) at 37°C with 5% CO_2_. Cells at 80–90% confluence were treated in a temperature-controlled water bath for 40 min at 43°C, and then cultured under normal conditions for 2, 6, 8, 12 and 24 h. The cells solely cultured under normal conditions were used as the control.

### Sample preparation and quantitation

The Tca8113 cells (2×10^7^) were collected and centrifuged at 228 × g for 5 min. The subsequent cell pellets were washed with phosphate-buffered saline (PBS) and then centrifuged at 228 × g for 5 min. This was repeated three times. The cell pellets were resuspended in 250 *μ*l DIGE lysis buffer containing 7 M urea, 2 M thiourea, 65 mM Tris, 4% CHAPS, 0.2% IPG buffer and a protease inhibitor cocktail (Roche Diagnostics GmbH, Mannheim, Germany), which was sonicated briefly on ice. The cell lysates were centrifuged at 15,600 × g for 20 min at 4°C. The supernatant was collected as the cell lysate, and the protein concentration was measured with the Bio-Rad Protein Assay (Bio-Rad Laboratories, Hercules, CA, USA) and adjusted to the same concentration for each sample.

### 2D-DIGE assay

Electrophoresis was conducted as described previously (29), and was performed with a model E-600 electrophoresis system (Amersham, Piscataway, NJ, USA). The pH of the protein samples was set at 8.0–9.0, and the samples were diluted to 5 *μ*g/*μ*l. All samples were mixed to equal amounts and aliquoted at 50 *μ*g/10*μ*l, which was then used as the internal standard. A total of 50 *μ*g sample was labeled with 400 pmol/*μ*l Cy3 and Cy5, respectively. The internal standard was labeled with 400 pmol/*μ*l Cy2. The three types of labeled samples were mixed evenly and the proteins were separated by isoelectric focusing electrophoresis (IFE), with a pH gradient of 3.0–10.0 on a 13-cm non-linear strip. The IFE conditions were as follows: 30 V for 12 h, 500 V for 1 h, 1,000 V for 1 h, 8,000 V for 8 h and 500 V for 4 h. Following isoelectric focusing, IPG strips were placed in equilibrium buffer and the second electrophoresis stage [12.5% sodium dodecyl sulfate-polyacrylamide gel electrophoresis (SDS-PAGE)] was subsequently conducted. The SDS-PAGE conditions were 15 mA for 20 min and 30 mA until the bromophenol blue front reached the bottom of the gel.

### Gel scanning and software analysis

The gel pattern was scanned with a Typhoon scanner (GE Healthcare Biosciences, Pittsburgh, PA, USA) at wavelengths of 488, 532 and 633 nm for Cy2, Cy3 and Cy5, respectively, and optimized by ImageQuant software (GE Healthcare Biosciences). Differential points were analyzed by DeCyder™ 2D software, version 6.5 (GE Healthcare).

### Protein identification

The gels were stained with Coomassie brilliant blue R350 (Amersham). The differential points were removed and the stain was removed in NH_4_HCO_3_/30% ACN buffer, prior to being digested in trypsin for 20 h. Peptides were extracted, desalted with a ZipTip (Millipore Corporation, Billerica, MA, USA) and analyzed by a 4800 Plus MALDI TOF/TOF™ analyzer (Applied Biosystems, Foster City, CA, USA). The differentially expressed proteins were identified by software analysis, as well as SwissProt and NCBInr database searches.

### Western blot analysis

Equal amounts of protein were separated by 10% SDS-PAGE and electroblotted onto Immobilon-P membranes (Millipore Corporation). The membranes were incubated with the primary antibody at 4°C overnight and then incubated with horseradish peroxidase-conjugated secondary antibody at room temperature for 1 h. Following washing with 1X Tris-buffered saline with 0.1% Tween-20 (TBST), the membranes were visualized by chemiluminescence (Pierce, Biotechnology, Inc., Rockford, IL, USA). The relative protein content is the ratio of the test protein gray scale value in contrast with the α-tubulin gray scale value, measured by ImageJ software (version 2.1.4.6; National Institutes of Health, Bethesda, MD, USA).

### Statistical analysis

Differences in the protein expression profile were analyzed by DeCyder 2D software, version 6.5, and a Student’s t-test was applied. Differences among the 6 groups were compared using a one-way ANOVA. P<0.01 was considered to indicate a statistically significant difference.

## Results

### 2D-DIGE gel pattern and analysis of differential protein points

**Figure 1.**
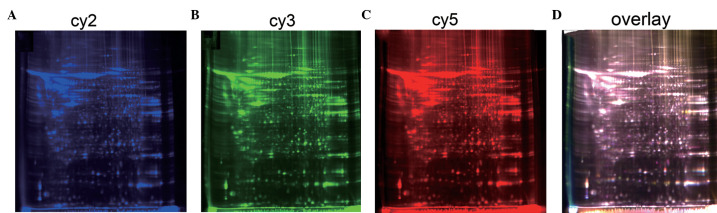
Protein fluorescence and overlay images. Protein samples were labeled with Cy2, Cy3 and Cy5, which were separated by two-dimensional differential in gel electrophoresis (DIGE), and the respective blue, green and red gel images were captured for each dye. (A) The image of the protein sample (labeled with Cy2) of the Tca8113 cells cultured under normal conditions. (B) The image of the protein sample (labeled with Cy3) of the Tca8113 cells treated for 40 min at 43°C and then cultured under normal conditions for 2 h. (C) The image of the protein sample (labeled with Cy5) of the Tca8113 cells treated for 40 min at 43°C and then cultured under normal conditions for 6 h. (D) Overlay of images A, B and C.

**Figure 2.**
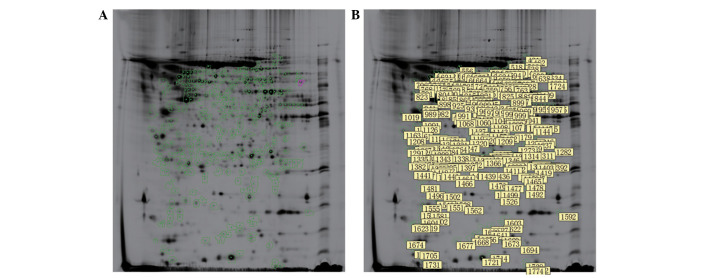
Differentially expressed protein points. The gel images were analyzed by DeCyder 2D software, version 6.5, and the data were compared by a one-way statistical analysis, which identified 107 differentially expressed proteins. (A) The 107 differentially expressed protein points. (B) The master numbers of the 107 differentially expressed protein points.

The Tca8113 cells were incubated for 40 min in a water bath at 43°C, and subsequently transferred to normal culture conditions for 2, 6, 8, 12 and 24 h. Protein samples were labeled with Cy2, Cy3 and Cy5, which were separated by 2D-DIGE, and the respective blue, green and red gel images were captured for each dye (Fig. 1). The gel images were analyzed by DeCyder 2D software, version 6.5, and the data were compared by a one-way statistical analysis, which identified 107 differentially expressed proteins (P=0.000; Fig. 2).

### Differential protein point identification and dynamic expression analysis

A total of 107 differential protein points were detected by tandem mass spectroscopy and analyzed using the SwissProt and NCBInr databases, which identified 57 types of proteins (Tables I and II). The early period was considered to be within 8 h of hyperthermic treatment, while 8–24 h post-treatment was considered to be the middle to late period. Protein expression levels that were higher in the hyperthermia-treated cells compared with the controls were considered to be upregulated, while proteins with lower expression levels were considered to be downregulated. The dynamic expression changes in 57 proteins, at 2, 6, 8, 12 and 24 h after hyperthermic treatment, were characterized. In total, 44 proteins were demonstrated to be upregulated and 13 proteins were shown to be downregulated.

**Figure 3.**
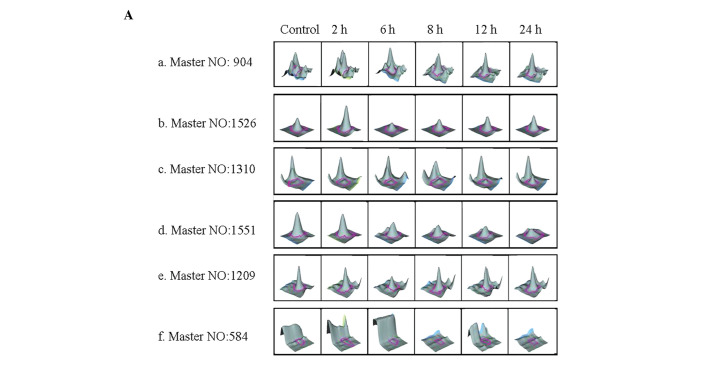

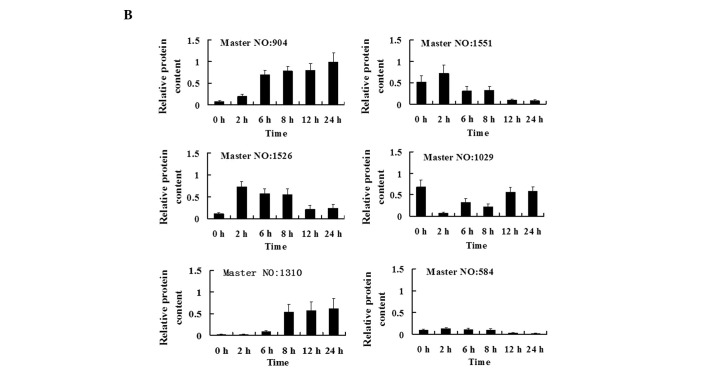
Dynamic changes in the expression levels of the differentially expressed proteins. (Aa). Master no. 904; heat shock 70 kDa protein 1 was upregulated continuously. (Ab) Master no. 1526; cofilin-1 was upregulated during the early time period. (Ac) Master no. 1310; phosphoglycerate mutase 1 was upregulated during the middle to late time period. (Ad) Master no. 1551; stathmin 1/oncoprotein 18 was downregulated continuously. (Ae) Master no. 1209; Δ(3,5)-Δ(2,4)-dienoyl-CoA isomerase, mitochondrial was downregulated during the early time period. (Af) Master no. 584; lamin-A/C was downregulated during the middle to late time period. (B) The relative content of representative proteins to α-tubulin following heating for 0, 2, 6, 8, 12 and 24 h, respectively. Data represent the mean from three independent experiments (bars, mean ± standard deviation).

Among the 44 upregulated proteins, 27 were upregulated continuously, 8 were upregulated during the early time period and 9 were upregulated during the middle to late time period (Table I; Fig. 3Aa–c, a representative figure of the protein expression changes during the three time periods). Among the 13 downregulated proteins, 5 were downregulated continuously, 6 were downregulated during the early time period and two were downregulated during the middle to late time period (Table I; Fig. 3Ad–f).

**Figure 4.**
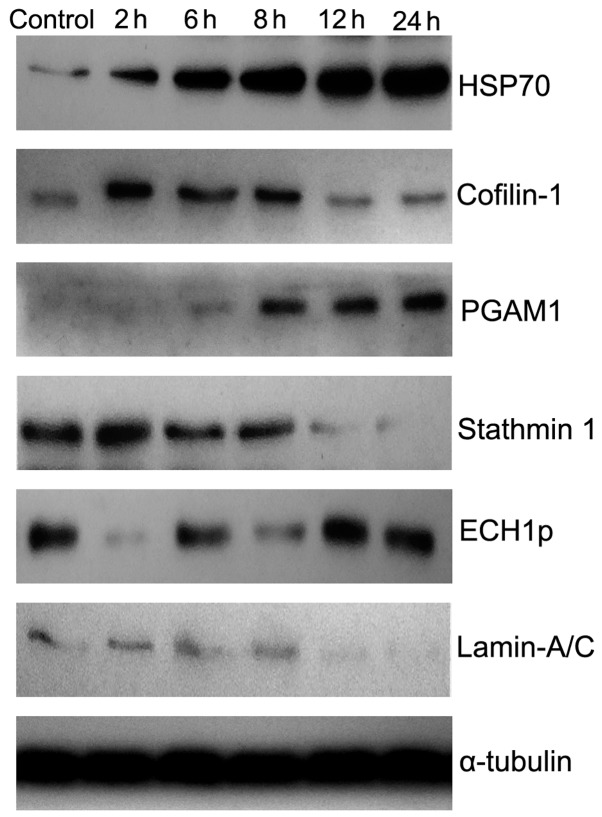
Western blot analysis of differentially expressed proteins following hyperthermia treatment. Heat shock 70 kDa protein (HSP70) was upregulated continuously within 24 h following hyperthermia treatment, cofilin-1 was upregulated during the early time period and phosphoglycerate mutase 1 (PGAM1) was upregulated during the middle to late time period. While stathmin 1 was downregulated continuously, Δ(3,5)-Δ(2,4)-dienoyl-CoA isomerase, mitochondrial (ECH1p) was downregulated during the early time period and lamin A/C was downregulated during the middle to late time period.

These protein expression profiles were further confirmed by western blot analysis (Fig. 4). The relative content of these proteins, following heating for 0, 2, 6, 8, 12 and 24 h, are shown in Table III and Fig. 3B (P= 0.000). Therefore, the results demonstrated that protein expression changed in a dynamic manner during hyperthermia-induced Tca8113 cell apoptosis.

## Discussion

The present study investigated protein expression differences in Tca8113 cells following hyperthermia, utilizing fluorescent differential display 2D gel electrophoresis. Changes in expression level were observed in a total of 107 proteins (Fig. 2B), and 57 of these proteins were identified by mass spectrometry analysis. The expression of these proteins varied over different time periods following hyperthermia treatment (Tables I and II) according to the following profiles: 27 were upregulated and 5 were downregulated continuously; 8 were upregulated and 6 were downregulated during the early time period; and 9 were upregulated and 2 were downregulated during the middle to late time period. These results demonstrated that the protein types and expression levels were each upregulated and downregulated during hyperthermia-induced Tca8113 cell apoptosis. These findings suggest that hyperthermia-induced Tca8113 cell apoptosis is controlled by multiple factors, which include time and regulatory proteins.

Overall, the proteins were grouped into five functional categories. The first functional category consisted of energy metabolism-related proteins. This category included the following proteins: Glyceraldehyde-3-phosphate dehydrogenase (GAPDH); α-enolase; inorganic pyrophosphatase; galactokinase; flavin reductase; phosphoglycerate kinase 1; pyridoxine-5′-phosphate oxidase; ATP synthase subunit d, mitochondrial; 26S proteasome non-ATPase regulatory subunit 14; protein disulfide-isomerase A3 (PDIA3); glutathione S-transferase P; PGAM1; 3-hydroxyacyl-CoA dehydrogenase type-2 isoform 1; crystal structure of Ufc1, the Ufm1-conjugating enzyme 1, chain A; pyruvate kinase isozymes M1/M2 (PKM1/2); and peroxiredoxin-4, -6 and -2. The levels of these energy metabolism-related proteins increased following hyperthermic induction. By contrast, the levels of the following energy metabolism-related proteins decreased: ATP synthase subunit α, mitochondrial; fructose-bisphosphate aldolase A (ALDOA); mitochondrial succinyl-CoA:3-ketoacid-coenzyme A transferase 1; D-3-phosphoglycerate dehydrogenase; argininosuccinate synthase, mitochondrial; Δ(3,5)-Δ(2,4)-dienoyl-CoA isomerase, mitochondrial (ECH1p); and hydroxysteroid (17-β) dehydrogenase 10 (HSD17B10). The changes in the expression levels of metabolism-related proteins may lead to impaired tumor cell substance metabolism and energy metabolism, which subsequently affect cell survival. Moreover, it has been demonstrated that certain proteins involved in the regulation of cell apoptosis (with the exception of metabolic proteins, such as GAPDH) may be induced to undergo nuclear translocation by ROS, and that increased GAPDH levels are a necessary step for apoptosis (30). For example, PKM2 initiates cell apoptosis by nuclear translocation through a caspase-independent pathway (31). Furthermore, argininosuccinate synthase levels correlate with Bcl-2 levels, and drug-induced cell apoptosis may be enhanced by downregulating argininosuccinate synthase expression (32). In addition, PGAM1 is overexpressed in multiple types of tumors and is involved in tumor formation; it has therefore been suggested that liver cancer cell apoptosis may be induced by the inhibition of PGAM1 (33). Moreover, the increased expression of peroxiredoxin II facilitates tumor cell survival (34), while peroxiredoxin IV inhibits radiation-induced tumor cell apoptosis (35). In the present study, the up- or downregulation of these aforementioned proteases after different time periods following hyperthermic treatment may have been involved in the regulation of apoptosis.

Cytoskeleton-related proteins are another of the five functional categories of proteins. This category includes: Actin, cytoplasmic 2; F-actin-capping protein subunit β; cofilin-1; tubulin β chain; keratin, type I cytoskeletal 10 (CK-10); keratin, type II cytoskeletal 1, 7 and 8 (CK-1,7 and 8); protein 4.1, isoform 3; and far upstream element-binding protein 1 (FBP1). Such proteins were upregulated after different time periods following hyperthermic treatment. The following three cytoskeleton-related proteins were downregulated: Lamin-A/C; vimentin and stathmin 1 (oncoprotein 18). These cytoskeletal proteins are involved in formation of the cell cytoskeleton (involving actin, microtubules, intermediate filaments and the microbeam network) in order to maintain cellular integrity, which is associated with tumorigenesis and the regulation of apoptosis (36–40). Changes in the expression levels of cytoskeleton-related proteins indicate damage to the cytoskeleton, which consequently affects the cell morphology and function. Cytoskeletal proteins have also been demonstrated to be involved in the regulation of apoptosis. Kouzu *et al* revealed that CK8 is involved in inhibiting several types of drug-induced apoptosis (41). Additionally, inhibiting far upstream element binding protein 1 (FBP1) has been shown to increase hepatocellular carcinoma (HCC) sensitivity towards apoptotic stimulation, which therefore inhibits cell proliferation (42). Furthermore, stathmin has been demonstrated to be downregulated upon the induction of hyperthermia, which also inhibits cell proliferation (43) and results in stathmin phosphorylation and dysfunctional microtubule assembly. Overall, these changes have been demonstrated to induce Jurkat cell apoptosis (44). The present study results indicated that within 24 h of hyperthermic induction, changes in the expression levels of a variety of cytoskeletal proteins became evident. In particular, changes were observed in the expression levels of stathmin and vimentin, which are proteins associated with tumor growth, while the tumor cell invasion and tumorigenesis dramatically decreased. Therefore, the results indicated that different cytoskeletal proteins are involved in the regulation of apoptosis after different time periods following hyperthermia.

Another of the functional protein categories was that of chaperones. This category included the following proteins: HSP70-1; heat shock cognate 71 kDa protein (HSPA8); HSP β-1 (HSPB1/HSP27); PDIA3; T-complex protein 1 subunit ζ; and 60 kDa HSP, mitochondrial (HSPD1). Such proteins were upregulated following the induction of hyperthermia. The main function of the chaperones is to maintain cell survival; HSP family members regulate mitochondrial pathway-mediated apoptosis and the death receptor-mediated apoptotic pathway. By blocking the hyperthermia-induced apoptotic pathway, HSPs are able to consequently inhibit apoptosis (22–25,28). In addition, T-complex protein 1 subunit ζ participates in the assembly of the cytoskeletal proteins actin and tubulin. Moreover, protein disulfide-isomerase A3 is located in the endoplasmic reticulum lumen and acts as a component of the calnexin/calreticulin chaperone complex. Therefore, this protein plays a significant role in calcium homeostasis and regulates free Ca^2+^, which is able to activate related enzymes and promote apoptosis (15,16). The present study results demonstrated that HSPs are upregulated within 24 h following hyperthermia, which may result in the regulation of apoptosis through different apoptotic signaling pathways. As PDIA3 and T-complex protein 1 subunit ζ were also upregulated following hyperthermia, we therefore hypothesized that these two proteins may participate in the regulation of hyperthermia-induced apoptosis.

Transcription factors and protein-synthesis related proteins comprise another of the functional protein categories. This particular category included the following proteins: Scavenger mRNA-decapping enzyme DcpS; crystal structure of Ufc1, the Ufm1-conjugating enzyme 1, chain A; heterogeneous nuclear ribonucleoprotein H; proteasome activator complex subunit 1; deoxyuridine 5′-triphosphate nucleotidohydrolase, mitochondrial (dUTPase); human eukaryotic translation initiation factor 1A (eIF1A), chain A; and splicing factor U2AF 65 kDa subunit (U2AF65). These proteins were upregulated after different time periods following hyperthermic treatment. By contrast, eIF5A-1 and the crystal structure of the human eIF5A, chain A were downregulated. It has been confirmed that certain transcription factors and protein synthesis-related proteins are involved in the regulation of apoptosis. For example, the overexpression of heterogeneous nuclear ribonucleoprotein H (hnRNP H) has been shown to partially counteract apoptosis induced by etoposide, and also to block mammalian STE20-like protein kinase 2 (MST2; proapoptotic MST2 kinase)-mediated apoptosis in cancer cells (45). In addition, eIF5A is able to regulate p53-dependent apoptosis by regulating p53 activity in COS-7 cells (46), while splicing factor U2AF65 subunit participates in Fas-mediated apoptotic regulation (47). Overall, these proteins are up- and downregulated following the induction of hyperthermia, and thus may contribute to hyperthermia-induced apoptotic regulation.

The final functional protein category comprised the cell division- and proliferation-related proteins, including prohibitin, mitotic checkpoint protein BUB3 and BolA-like protein 2, which were upregulated following hyperthermia treatment. However, one of the proteins in this category, proliferation-associated protein 2G4, was downregulated. Prohibitin is a multifunctional protein that is involved in the following processes: i) the regulation of cell signaling, apoptosis and survival (48); ii) the regulation of cell cycle progression and function as an anti-proliferative protein that is considered to be a tumor suppressor; and iii) the inhibition of cell division and tumor growth (49). The present study results indicated that prohibitin is upregulated continuously following hyperthermia treatment. As a tumor suppressor, this protein may participate in the promotion of hyperthermia-induced Tca8113 cell apoptosis.

The present study demonstrated that the expression levels of 57 proteins were dramatically regulated in Tca8113 cells within 24 h of hyperthermia treatment. The altered proteins were grouped within the following functional classes: energy metabolism-related enzymes, cytoskeleton-related proteins, chaperones, transcription factors and protein synthesis-related proteins and cell division- and proliferation-related proteins. However, no changes were detected in the expression levels of the hyperthermia-induced apoptosis-related proteins that had been previously identified to exhibit such changes, such as p53, Bax, Bak, caspases, cytochrome *C* (9,17–20), apoptosis-inducing factor (AIF), Bcl-2 and Mcl-1, which may have been due to the low expression of such proteins. Moreover, certain proteins were not detectable by 2D-DIGE. The present study found that the following pro-apoptotic proteins were upregulated: GAPDH, PKM2, eIF5A, prohibitin and mitotic checkpoint protein BUB3. In addition, certain anti-apoptotic proteins were downregulated, including stathmin, vimentin and argininosuccinate synthase. Notably, certain anti-apoptotic proteins were upregulated, including FBP1, hnRNP H, the peroxiredoxins, the HSPs and PGAM1. The expression of 57 proteins was either upregulated or downregulated within 24 h of hyperthermic treatment. These findings indicate that hyperthermia-induced Tca8113 cell apoptosis is controlled by multiple factors, which include time and regulatory proteins; however, the underlying mechanisms for these changes require further investigation.

## Figures and Tables

**Table I.** Upregulation of 44 proteins during the 24 h following hyperthermia.

Upregulated continuously	Upregulated early	Upregulated at a middle to late period
α-enolase	BolA-like protein 2	Peroxiredoxin-6
Actin, cytoplasmic 2	Cofilin-1	Phosphoglycerate mutase 1
Crystal structure of Ufc1, the Ufm1-conjugating enzyme 1, chain A	Deoxyuridine 5′-triphosphate nucleotidohydrolase, mitochondrial	Human eukaryotic translation initiation factor 1A, chain A
F-actin-capping protein subunit β	Glutathione S-transferase P	T-complex protein 1 subunit ζ
26S proteasome non-ATPase regulatory subunit 14	Far upstream element-binding protein 1	Splicing factor U2AF 65 kDa subunit
Glyceraldehyde-3-phosphate dehydrogenase	Peroxiredoxin-2	Scavenger mRNA-decapping enzyme DcpS
Heat shock protein β-1	Pyruvate kinase isozymes M1/M2	Keratin, type II cytoskeletal 8
Inorganic pyrophosphatase	Tubulin β chain	60 kDa heat shock protein, mitochondrial
Heterogeneous nuclear ribonucleoprotein H		3-hydroxyacyl-CoA dehydrogenase type-2 isoform 1
Mitotic checkpoint protein BUB3		
Phosphoglycerate kinase 1		
hCG15971, isoform CRA_b		
Protein disulfide-isomerase A3		
Proteasome activator complex subunit 1		
Peroxiredoxin-4		
Serum albumin		
Flavin reductase		
Galactokinase		
Prohibitin		
Protein 4.1, isoform 3		
Pyridoxine-5′-phosphate oxidase		
ATP synthase subunit d, mitochondrial		
Heat shock cognate 71 kDa protein		
Heat shock 70 kDa protein 1		
Keratin, type I cytoskeletal 10		
Keratin, type II cytoskeletal 7		
Keratin, type II cytoskeletal 1		

**Table II.** Downregulation of 13 proteins during the 24 h following hyperthermia.

Downregulated continuously	Downregulated early	Downregulated at a middle to late period
ATP synthase subunit α, mitochondrial	Argininosuccinate synthase, mitochondrial	Hydroxysteroid (17-β) dehydrogenase 10
Crystal structure of the human eukaryotic translation initiation factor 5A, chain A	D-3-phosphoglycerate dehydrogenase	Lamin-A/C
Fructose-bisphosphate aldolase A	Δ(3,5)-Δ(2,4)-dienoyl-coenzyme A isomerase, mitochondrial	
Vimentin	Eukaryotic translation initiation factor 5A-1	
Stathmin 1/oncoprotein 18	Proliferation-associated protein 2G4	
	Mitochondrial succinyl-CoA:3-ketoacid-coenzyme A transferase 1	

**Table III.** The relative content of representative proteins following heating for different time periods.

Master no.	0 h	2 h	6 h	8 h	12 h	24 h
904	0.07±0.02	0.19±0.05	0.69±0.11	0.78±0.11	0.80±0.15	0.99±0.20
1526	0.11±0.03	0.73±0.12	0.57±0.11	0.55±0.13	0.21±0.09	0.24±0.08
1310	0.02±0.01	0.02±0.01	0.09±0.02	0.54±0.17	0.57±0.20	0.62±0.23
1551	0.52±0.14	0.72±0.19	0.31±0.10	0.33±0.09	0.10±0.02	0.08±0.03
1029	0.68±0.17	0.07±0.02	0.32±0.09	0.22±0.07	0.56±0.11	0.59±0.09
584	0.10±0.02	0.13±0.03	0.11±0.03	0.10±0.03	0.03±0.01	0.02±0.01

Relative to α-tubulin. All data are presented as mean ± SD.
